# Universal field matching in craniospinal irradiation by a background‐dose gradient‐optimized method

**DOI:** 10.1002/acm2.12204

**Published:** 2017-11-07

**Authors:** Erik Traneus, Nicola Bizzocchi, Francesco Fellin, Barbara Rombi, Paolo Farace

**Affiliations:** ^1^ RaySerach Laboratories Stockholm Sweden; ^2^ Proton therapy Unit Hospital of Trento Trento Italy

**Keywords:** craniospinal irradiation, field junction, proton therapy, VMAT

## Abstract

**Purpose:**

The gradient‐optimized methods are overcoming the traditional feathering methods to plan field junctions in craniospinal irradiation. In this note, a new gradient‐optimized technique, based on the use of a background dose, is described.

**Methods:**

Treatment planning was performed by RayStation (RaySearch Laboratories, Stockholm, Sweden) on the CT scans of a pediatric patient. Both proton (by pencil beam scanning) and photon (by volumetric modulated arc therapy) treatments were planned with three isocenters. An ‘in silico’ ideal background dose was created first to cover the upper‐spinal target and to produce a perfect dose gradient along the upper and lower junction regions. Using it as background, the cranial and the lower‐spinal beams were planned by inverse optimization to obtain dose coverage of their relevant targets and of the junction volumes. Finally, the upper‐spinal beam was inversely planned after removal of the background dose and with the previously optimized beams switched on.

**Results:**

In both proton and photon plans, the optimized cranial and the lower‐spinal beams produced a perfect linear gradient in the junction regions, complementary to that produced by the optimized upper‐spinal beam. The final dose distributions showed a homogeneous coverage of the targets.

**Discussion:**

Our simple technique allowed to obtain high‐quality gradients in the junction region. Such technique universally works for photons as well as protons and could be applicable to the TPSs that allow to manage a background dose.

## 1 INTRODUCTION

In craniospinal irradiation the large target volume needs complex treatment planning. With the exception of helical tomotherapy treatments, it entails setting multiple isocenters and matching a large number of fields to obtain satisfactory plans.^1^, ^2^ The field junction area is a critical region, because under‐dosage may compromise tumor control, while over‐dosage may increase the risk of serious radiation‐induced late effects, e.g. spinal cord radionecrosis.

Field‐junctions planning techniques have often been investigated over the course of the years. The irradiation technique evolved from conformal to intensity modulated and recently pencil beam scanning proton therapy. In line with these developments, the techniques for planning field junction evolved from the moving junction techniques, often referred to as field feathering, to the method exploiting the potential of dose modulation and inverse planning.

It has recently been shown that the most robust field‐junction to setup errors is obtained by the so‐called gradient‐optimized methods, i.e. by producing a slow, linear and complementary dose gradient at the beam edges in the overlapping region between adjacent beams.^3^, ^4^ These methods showed a reduced sensitivity to longitudinal setup errors compared to the conventional feathering methods.^5^ Dose deviations increased linearly with setup errors, and the magnitude of increase depended on the junction length,^3^ i.e. the ratio between the dose error and the prescribed dose was proportional to the ratio between the setup error and the junction length.^6^

The gradient‐optimized methods are overcoming the traditional multiple‐junction shifts, and no standardized planning method exists. In the following paragraphs, we describe a new gradient‐optimized technique to plan field junction, based on the use of a background dose. Typically, a background dose is the dose resulting from a previous irradiation, when it is managed by the treatment planning systems (TPS) to optimize the successive re‐irradiation over the previous dose distribution. In our approach, the background dose was modeled a priori to produce a background gradient in the junction area, and it is used to guide inverse planning of the treatment beams.

## 2 METHODS AND RESULTS

TPS simulations were performed in RayStation (RaySearch Laboratories, Stockholm, Sweden) on the CT scans of a pediatric patient. Both a proton treatment by pencil beam scanning and a photon treatment by volumetric modulated arc therapy (VMAT) were planned with three isocenters. Dose prescription was 36 GyE in 20 fractions. Proton treatment was planned by two opposed oblique‐lateral cranial fields plus two additional postero‐anterior spinal beams. VMAT was planned by full arc spanning from −180 to 179 degrees around the patient with a 4 degree gantry spacing.

**Figure 1:**
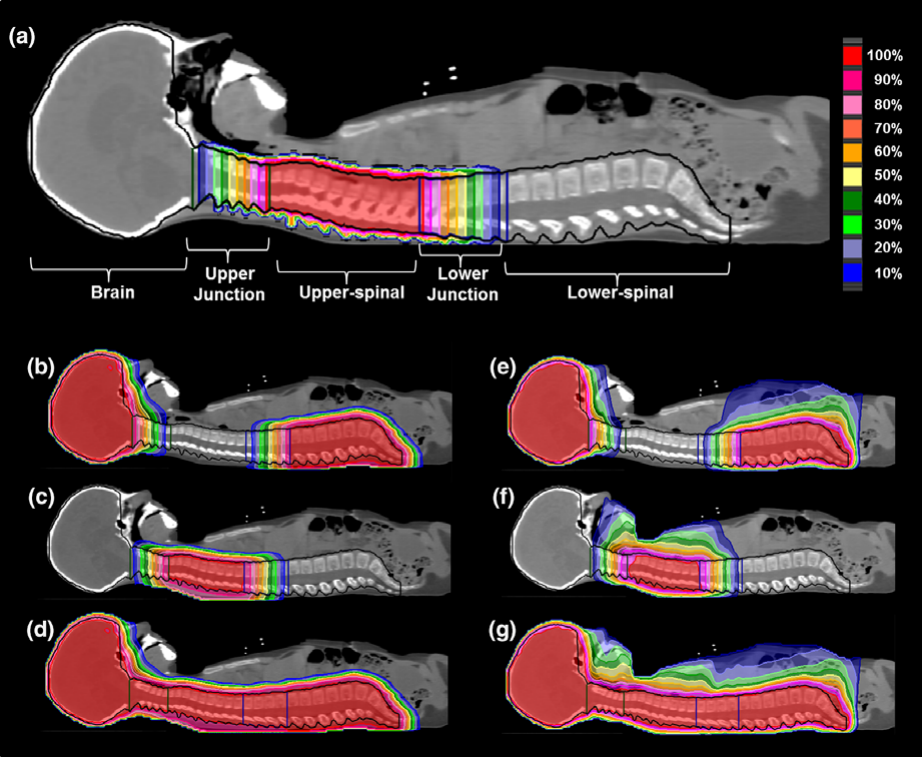
Field junction by the background dose technique (a) with protons by pencil beam scanning (on the left, b–d) and with photons by VMAT (on the right, e–g). The target was divided into five volumes: brain, upper‐junction, upper‐spinal, lower‐junction, and lower‐spinal. Dose distributions of the optimized brain and lower‐spinal fields (b, e), of the optimized upper‐spinal field (c, f) and total dose distributions (d, g) are shown.

**Figure 2:**
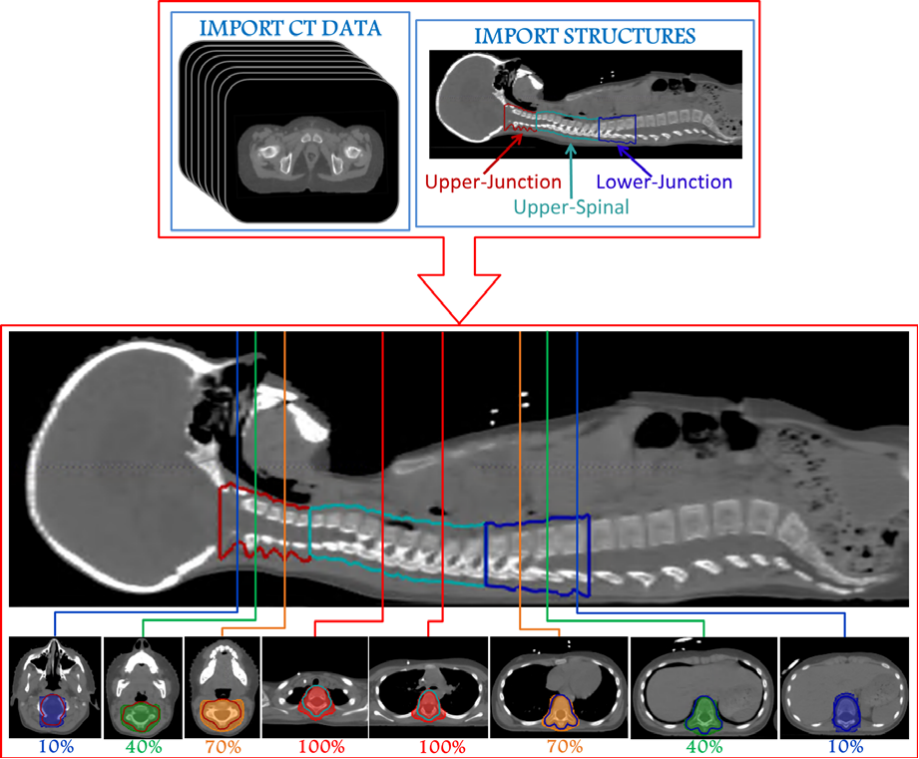
Background dose generation. Patient CT data and structures are exported from the TPS and uploaded into a numerical computing environment (MATLAB ^®^, The MathWorks.inc, Natick, MA, USA). To generate the background dose, three contoured volumes were considered: upper‐junction, upper‐spinal and lower‐junction. In each CT slice containing an upper‐spinal contour, the voxels of the background dose matrix inside the contour were assigned by a uniform value equal to 100% of the prescription dose. In each CT slice containing an upper‐ or lower‐junction contour, the voxels of the background dose matrix inside the contour were assigned by a uniform value, which depended on the position along the cranio‐caudal direction, to produce a perfect dose gradient along the upper‐ and lower‐junction PTVs [see Fig. 1(a)].

The planning treatment volumes (PTV) were delineated as in Fig. 1(a). Brain, upper‐spinal and lower‐spinal PTVs were separated by the upper‐ and lower‐junction PTVs. The background dose was designed to cover the upper‐spinal PTV by the prescription dose and to produce a perfect dose gradient along the upper‐ and lower‐junction PTVs [Fig. 1(a)]. The cost function objectives used during the optimization workflow are reported in Table 1. The background dose was produced *in silico* using a numerical computing environment (MATLAB^®^, The MathWorks.inc, Natick, MA, USA). Patient CT data and structures are exported from the TPS in DICOM format and then uploaded into the numerical computing environment. The background dose was built as a three dimensional matrix with the same size of the CT scan (Fig. 2). To generate it, three contoured volumes were considered: upper‐junction, upper‐spinal and lower‐junction. In each CT slice containing an upper‐spinal contour, the voxels of the background dose matrix inside the contour were assigned by a uniform value equal to 36 GyE (= 100% of the prescription dose). Each CT slice containing an upper‐junction contour was numbered with a number N_s_, being in the more cranial slice N_s_ = 1 and in the more caudal slice N_s_ = N. Then in each CT slice the voxels of the background dose matrix inside the upper‐spinal contours were assigned by a uniform value equal to Ns/N × 36 GyE. The same procedure was applied for the CT slices containing a lower‐junction contour, but using an inverse CT slice numbering along the cranio‐caudal direction. Each voxel of the dose matrix outside the upper‐junction, upper‐spinal, and lower‐junction volumes was assigned by zero value. The obtained dose matrix was then interpolated to fit to the spatial resolution of a TPS computed dose matrix (typically 0.2 × 0.2 × 0.2 cm^3^) and then exported in DICOM format to be uploaded by the TPS as in Fig. 1(a). In the future, a specific script can be realized to produce the same dose distribution by using RaySearch scripting tools. The background dose covered upper‐spinal PTV by the prescription dose to avoid (by using a max‐dose objective, see Table 1) in the optimization of the brain and lower‐spinal beams that any undesired spot was delivered to the upper‐spinal PTV. While keeping such dose as a background, in a first step the brain and the lower‐spinal beams were planned, by inverse optimization, in order to obtain dose coverage of the respective PTVs and of the junction volumes. To produce a perfect dose gradient along the upper‐ and lower‐junction PTVs, uniform doses objectives (with dose set at the prescription dose, see Table 1) were applied, so that the dose produced by the optimized beams plus the background dose was uniform at the end of the optimization. The dose distributions obtained after removal of the background dose are shown in Fig. 1(b) (proton) and Fig. 1(e) (photon). In the junction PTVs, the dose distribution was characterized by a complementary dose gradient with respect to the original background dose. Finally, in the second step of the optimization procedure (Table 1), the upper‐spinal field was inversely planned using the dose obtained by the previously optimized brain and lower spinal fields as background dose. The dose distributions of the optimized upper‐spinal fields are reported in Figs. 1(c) and 1(f). The final dose distributions [Figs. 1(d) and 1(g)] showed a homogeneous coverage of the PTVs.

**Table 1:** Planning objectives and workflow

Step #	Optimized beam(s)	Applied background dose	Cost function objectives^a^
1	Brain; Lower‐spinal	Background dose produced *in silico* and shown in Fig. 1(a)	Brain PTV: uniform dose 36 GyE; Upper‐junction PTV: uniform dose 36 GyE; Upper‐spinal PTV: max dose 36GyE Lower‐junction PTV: uniform dose 36 GyE; Lower‐spinal PTV: uniform dose 36 GyE
2	Upper‐spinal	Dose distribution obtained by the previous step 1	Brain PTV: max dose 36 GyE; Upper‐junction PTV: uniform dose 36 GyE; Upper‐spinal PTV: uniform dose 36 GyE Lower‐junction PTV: uniform dose 36 GyE; Lower‐spinal PTV: max dose 36 GyE

a The target was divided into five volumes [as shown in Fig. 1(a)]: brain, upper‐junction, upper‐spinal, lower‐junction and lower‐spinal.

## 3 DISCUSSION

It has been demonstrated that gradient‐dose junctions reduce the risk of dose overlap or underlap owing to field setup errors compared with the conventional feathering technique.^5^ Feathering can in principle be as robust if enough (i.e. very many) “feathers” are used, but the feathering planning and quality assurance process would require much more work to be practicable. For a given junction planned by a gradient method, deviations increased linearly with setup error, and the magnitude of increase depended on the junction length. Given the same setup errors, a larger gradient length reduced the associated dose deviations.^3^ Even though in the gradient method there is some anatomical limitation to the upper junction length, potential dose deviations in the upper and lower junctions were small after kilovoltage alignment.^4^

After the first studies where adjacent beams were intentionally overlapped to alleviate the issues of beam edge matching,^7^, ^8^ different approaches were proposed to properly obtain dose gradient in the junction area. In Myers et al.,^9^ using VMAT, the junction area of the target where the two arcs overlapped was contoured as four, equal‐in‐length sections. These sections were used to guide inverse planning and shape the dose gradients across the field junction. Using proton pencil beam scanning, in Lin et al.^3^ the target was divided along the cranio‐caudal axis in four equally spaced segments and in Stoker et al.^10^ in four to ten equally sized segments to guide optimization. Using pencil beam scanning proton therapy, a different approach was recently proposed^4^, ^6^ that introduced an ancillary beam (used only to guide the inverse planning and not for dose delivery) to produce in the junction region a linear dose‐gradient along the cranio‐caudal axis.

In the comparison among different techniques, at least three different features should be considered: (a) quality (i.e. the uniform linearity) of the obtained gradient in all the junction area, (b) simplicity, to avoid cumbersome planning procedure and (c) applicability to different irradiation techniques (i.e. photon and protons) and different TPSs.

The techniques that segmented the targets to guide the planning of field junction were applicable to different irradiation modalities and in fact they were reported both for photons^9^ and protons.^3^ However, they suffer of cumbersome procedures and poor quality of the linear gradient when increasing the length of the junction area, resulting in step‐shaped gradients.^9^ To improve the quality of the junction, the number of segments should be increased with a consequent increase in plan complexity, and many volumes to be managed during optimization. On the other hand, the ancillary beam method^4^, ^6^ easily produced high quality gradients, but it is applicable only if a proton therapy machine equipped with pencil beam scanning is commissioned on the TPS.

The technique described in this short note could overcome the limitations of the other gradient‐optimized methods. In fact, it produced high‐quality gradients in a very simple way and it could be applicable for both protons and photons and therefore it can be considered ‘universal’. Furthermore, it can be implemented in all TPSs that allow to manage a background dose (for example in Eclipse™, Varian Medical Systems, Inc. where a similar technique has been used to create the split fields for large field IMRT planning).

## CONFLICT OF INTEREST

Erik Traneus reports being employed by RaySerach Laboratories (Stockholm, Sweden) during the conduct of the study and outside the submitted work. Nicola Bizzocchi, Francesco Fellin, Barbara Rombi, and Paolo Farace report no conflict of interest.
